# Reactor-Aware Machine Learning Coupled with Differential Evolution for Predicting and Optimizing Cumulative Methane Production from Agro-Industrial Waste Co-Digestion

**DOI:** 10.3390/foods15173161

**Published:** 2026-09-07

**Authors:** Juan Carlos DelaVega-Quintero, Jimmy Nuñez-Pérez, Marco Lara-Fiallos, Wendy Salazar

**Affiliations:** 1 Agroindustrial Products from Residues Research Group, Universidad Técnica del Norte, Ibarra 100104, Imbabura, Ecuador; 2 School of Agroindustry, Universidad Técnica del Norte, Ibarra 100104, Imbabura, Ecuador

**Keywords:** support vector regression, differential evolution, leave-one-reactor-out cross-validation

## Abstract

Anaerobic digestion of agro-industrial residues supports waste valorization and renewable-energy production, but reliable prediction requires validation that accounts for repeated measurements within reactors. This study compared 16 regression models for predicting cumulative methane production from digestion time and banana peel–sugarcane molasses composition using 5007 observations from seven batch reactors. Models were evaluated by leave-one-reactor-out cross-validation (LORO-CV). Radial-basis-function support vector regression (SVR-RBF; C = 10, gamma = “scale”, epsilon = 0.1) achieved the lowest pooled RMSE (118.09 NmL CH_4_), with R^2^ = 0.9482 and MAE = 75.75 NmL CH_4_, and was selected as the surrogate model. However, reactor-level Wilcoxon tests with Holm correction showed no significant differences between SVR-RBF and the other algorithms. Held-out-reactor R^2^ values ranged from −1.366 to 0.928, indicating heterogeneous generalization. Differential Evolution consistently identified approximately 100% banana peel and 0% molasses as the optimal composition. Across 70 runs, the median optimum was 310.10 h and 1433.25 NmL CH_4_. Bootstrap analysis placed 99% of composition optima at ≥99% banana peel, although uncertainty in optimal time was substantial. Kinetic benchmarking supported the slower, higher-volume methane production observed in complete banana-peel reactors. This boundary solution is therefore a model-supported candidate requiring experimental confirmation, not a universal co-digestion optimum.

## 1. Introduction

Anaerobic digestion (AD) is a complex biochemical process where microorganisms decompose organic matter in an oxygen-free environment, progressing through four sequential stages: hydrolysis, acidogenesis, acetogenesis, and methanogenesis [1,2]. Effective environmental management relies on the valorization of diverse organic residues including food waste, agricultural residues, and industrial by-products [3]. Specifically, industrial waste streams such as sugar-rich distillery stillage, whey, and molasses are produced in significant quantities and contain high organic content suitable for conversion into high-calorific energy sources like biomethane [4].

The co-digestion of biowaste for biomethane generation is an inherently complex and non-linear process that depends on numerous independent control factors [5]. Traditional kinetic models, such as the Anaerobic Digestion Model No. 1, frequently struggle with site-specific variations and demand extensive parameter tuning, often missing the non-linear interactions at play [6]. That is why artificial intelligence and machine learning (ML) have stepped in, capable of uncovering hidden relationships within complex, high-dimensional data without relying on predefined biochemical rules [7]. A range of techniques, from Artificial Neural Networks and Support Vector Machines to ensemble methods like Random Forest (RF), have already proven their worth in forecasting biogas production, monitoring system stability, and fine-tuning operational parameters [8].

For all its strengths, especially its remarkable flexibility and predictive power, AI is not without its hurdles. Many deep learning models suffer from the so-called “black box” issue, where poor interpretability undermines operator confidence and slows down regulatory approval [9]. What is more, these models are heavily dependent on the volume and quality of historical records, making them vulnerable to gaps in data or unreliable sensor readings [10]. Solid preprocessing becomes non-negotiable here: outliers must be rigorously spotted and removed, using tools like Z-score or interquartile range, while missing entries need careful imputation to avoid warping the training process [11]. Equally important is the need to guard against data leakage and overfitting, so that models genuinely generalize to fresh data rather than just memorizing old examples [12].

Machine-learning and evolutionary optimization methods have previously been applied to anaerobic digestion, Yildirim and Ozkaya compared five machine-learning algorithms using operational data from an industrial-scale anaerobic digestion plant [13], whereas Ghazizade Fard and Koupaie evaluated several tree-based and nonlinear algorithms for predicting sludge solubilization and methane production following hydrothermal pretreatment [14]. Karamichailidou et al. had already combined radial-basis-function networks with Differential Evolution for modelling biogas production from anaerobic wastewater treatment plants [15]. Therefore, neither SVR-RBF nor its coupling with Differential Evolution constitutes an algorithmic novelty in the present study. In contrast to these previous applications, the contribution of the present work lies in applying a reactor-aware validation and uncertainty framework to repeated batch trajectories from the comparatively underexplored banana peel–sugarcane molasses system. This framework combines leave-one-reactor-out validation, reactor-level statistical comparisons, fold-specific optimization, reactor-level bootstrap resampling, and kinetic benchmarking.

The research gap addressed in this study is instead experimental and validation-oriented. Previous investigations have examined banana peel digestion alone or with animal manure [16,17], whereas molasses digestion has generally been studied with manure, wastewater, or other buffering co-substrates [18]. Comparatively little information is available on the specific banana peel–sugarcane molasses system and on how its complete batch methane trajectories can be used for reactor-aware prediction and constrained composition optimization. Furthermore, random row-wise cross-validation can produce optimistic estimates when repeated time points from the same reactor are distributed between the training and validation sets. The present study therefore combines: (i) a feedstock-specific evaluation of banana peel and sugarcane molasses; (ii) leave-one-reactor-out validation; (iii) reactor-level paired statistical comparisons with multiplicity correction; (iv) grouped optimization and reactor-level bootstrap uncertainty analysis; and (v) kinetic-model benchmarking.

Accordingly, this study aimed to compare regression algorithms for predicting cumulative methane production from digestion time and substrate composition, select a model under leave-one-reactor-out validation, evaluate the stability and limitations of its predictions across independent reactors, and use the selected model as a surrogate objective for Differential Evolution within the experimentally defined domain.

## 2. Materials and Methods

### 2.1. Waste Collection and Preparation of the Substrate

Banana peels were collected from the wholesale market in Ibarra city, Ecuador, while molasses was acquired from the Ingenio Azucarero del Norte, a sugar mill in the same city. Sheep manure served as the inoculum for the AD process. Before utilization, both substrates were characterized: Chemical oxygen demand (COD) was determined using a Lovibond MD 100 COD colorimeter (Tintometer GmbH, Dortmund, Germany), following the manufacturer’s operating procedure and the corresponding COD reagent/test protocol. Volatile solid was determined using APHA 2540 E [19]. Total solids (TS) was determined using APHA 2540 B [19]. Total carbohydrate content was determined using the phenol–sulfuric acid colorimetric method described by Dubois et al. [20], with absorbance measured at 490 nm. Elemental carbon (C), nitrogen (N), hydrogen (H), and sulfur (S) were determined using an Elementar Vario Macro Cube elemental analyzer (Elementar Analyse system GmbH, Dortmund, Germany). The C/N ratio was calculated as the quotient of the measured elemental carbon and nitrogen mass fractions. Characterization measurements were performed in triplicate, and results are expressed as mean ± standard deviation. The resulting physicochemical characteristics of banana peel, sugarcane molasses, and inoculum are summarized in Table 1. The feedstock’s pH was adjusted to 7.5 by adding sodium hydroxide solution as needed. The inoculum was prepared according to standard methods and filtered to eliminate large particles before being added to the bioreactors.

To eliminate impurities and harmful microorganisms, banana peels were first washed and then left to drain. Molasses was combined with water in a 1:10 ratio, after which it was blended with the inoculum and the peels, allowing for a 15-day adaptation phase. The reactors, with a nominal capacity of 500 mL, contained 400 mL of the different experiment mixtures.

### 2.2. Methane Measurement

Cumulative methane production was monitored using an Automatic Methane Potential Test System (AMPTS II, BPC Instruments AB, Lund, Sweden). Gas produced in each anaerobic reactor passed through the system’s CO_2_-absorption unit before reaching the gas-volume measuring device. The resulting cumulative methane volume was automatically recorded by the AMPTS II system and expressed as normalized milliliters of methane (NmL CH_4_). Gas volumes were automatically corrected by the instrument to dry-gas conditions at 0 °C and 1 atm (101.32 kPa) using internal temperature and pressure measurements. The experiment was conducted at a steady temperature of 36 °C for up to 16 days, agitation was automatically provided by the AMPTS II mixing system for 5 min every hour at 28 rpm to maintain homogeneous reactor conditions.

No inoculum-only blank reactors or positive substrate controls were included in the original experimental design. Consequently, the methane volumes were not corrected for endogenous methane production by the inoculum and were not normalized per unit of substrate VS. Therefore, the response variable used for kinetic and machine-learning analyses was cumulative methane volume (NmL CH_4_) from the complete inoculum–substrate system, rather than substrate-specific biochemical methane potential. Methane production was monitored until no further relevant methanogenic activity was observed. No predefined quantitative termination threshold was established in the original experimental protocol.

The substrate mixtures were formulated on an as-weighed mass basis (*w*/*w*), rather than on a TS-, VS-, or COD-normalized basis. A fixed substrate mass of 50 g was used in each reactor and combined with 100 g of previously adapted inoculum, resulting in an inoculum-to-substrate ratio of 2:1 on a mass basis. For the three configurations retained for subsequent machine-learning analysis, the substrate compositions were (Table 2): 0 g banana peel and 50 g sugarcane molasses (0/100), 12.5 g banana peel and 37.5 g sugarcane molasses (25/75), and 50 g banana peel and 0 g sugarcane molasses (100/0). The assays were conducted in nominal 500 mL AMPTS II reactors (BPC Instruments AB, Lund, Sweden), with a headspace volume of 100 mL configured in the AMPTS II software version ampts_2.1 (v1.2948). The samples were diluted with 50 mL of water.

Independent AMPTS II reactors were used as experimental replicates. Two reactor replicates were available for the 0/100 (0–100 R1 and 0–100 R2) and 25/75 (25–75 R1 and 25–75 R2) BP/SM conditions, whereas three reactors were available for the 100/0 condition (100–0 R1, 100–0 R2 and 100–0 R3). No separate technical replicates were performed; the high-frequency measurements collected over time within each reactor represent repeated temporal observations of the same experimental unit and were not considered independent replicates. No predefined acceptance threshold for between-reactor variability was established in the original experimental protocol.

The final dataset contained 5007 timestamped observations distributed across seven independent reactor trajectories in one AMPTS II batch campaign, each reactor vessel constituted one experimental unit. The number of observations differed among reactors because the duration of methanogenic activity varied among experimental conditions. One trajectory (100/0 R3) was prematurely truncated because of an operational recording failure of the AMPTS II system. This reactor was retained in the primary analysis because the measurements recorded before the failure represented valid experimental observations.

### 2.3. Machine-Learning Modelling and Process Optimization

The experimental database described in Section 2.2 was used for machine-learning modelling. The response variable was cumulative methane production (NmL CH_4_), whereas digestion time (h) and substrate composition were used as predictors. Because banana peel and sugarcane molasses percentages were complementary and summed to 100%, only banana peel percentage was included as an independent composition predictor; sugarcane molasses percentage was calculated as 100 − banana peel percentage. Records with missing cumulative methane values were excluded. No observations were removed through an outlier detection procedure.

Exploratory associations were assessed using Pearson correlation coefficients and univariate F statistics implemented through the f_regression function in Scikit-learn. Because the database contained repeated temporal measurements from the same reactors, these analyses were treated as descriptive summaries and not as independent observation inferential tests.

#### 2.3.1. Model Development, Validation, and Selection

Sixteen regression configurations were compared: linear regression (LR), ridge regression, radial-basis-function support vector regression (SVR-RBF), decision tree (DT), random forest (RF), gradient boosting regression (GBR), k-nearest neighbours (KNN), Gaussian process regression (GPR), and eight multilayer perceptron (MLP) configurations. Ridge regression used α = 1. The SVR-RBF model used C = 10, gamma = “scale”, and ε = 0.1. The DT used a maximum depth of 5 and a minimum of 5 observations per terminal leaf. The RF comprised 200 trees. The KNN model used five neighbours. The GPR combined a constant kernel with a Matérn kernel (ν = 1.5), used α = 10^−4^, normalized the response, and performed two optimizer restarts. The MLP used ReLU activation, the Adam optimizer, α = 10^−4^, a maximum of 5000 iterations, early stopping, a validation fraction of 0.20, and the following hidden-layer architectures: (10), (30), (50), (100), (50,25), (100,50), (50,50), and (100,50,25). A random seed of 42 was used for stochastic estimators. Predictor scaling was incorporated within the modelling pipeline where appropriate and was fitted exclusively using the training portion of each validation fold.

Model generalization was evaluated using leave-one-reactor-out cross-validation (LORO-CV). In each fold, all observations from one reactor were reserved for testing, while the model was trained using all observations from the remaining six reactors. Predictions from the seven held-out reactors were concatenated to calculate the coefficient of determination (R^2^), mean absolute error (MAE), mean squared error (MSE), and root mean squared error (RMSE). The model with the lowest pooled LORO-CV RMSE was selected, with MAE used as a secondary criterion in the event of a tie.

Statistical comparisons were conducted at the reactor level. For each algorithm and each held-out reactor, the MAE was calculated from that reactor’s predictions. The seven paired reactor-level MAEs of the selected model were compared with those of every alternative model using two-sided Wilcoxon signed-rank tests. The resulting *p*-values were adjusted across the 15 comparisons using Holm’s procedure, with an adjusted significance threshold of α = 0.05.

A grouped generalization diagnostic was additionally constructed for the selected model. For each LORO fold, training and held-out-reactor R^2^ and RMSE values were compared to identify heterogeneity among reactors and potential discrepancies between model fit and generalization. An exploratory sensitivity analysis was conducted by varying the hyperparameters. Each configuration was evaluated using the same LORO-CV procedure to assess the robustness of the model results to hyperparameter selection.

#### 2.3.2. Process Optimization

The seven fold-specific pipelines obtained during LORO-CV were used as surrogate objective functions. Differential Evolution was independently applied to each fold-specific pipeline using ten random seeds (1, 11, 21, 31, 41, 51, 61, 71, 81, and 91), resulting in 70 grouped optimization runs. The optimization domain was restricted to the experimental range: 0–388.75 h for digestion time and 0–100% for banana peel, with sugarcane molasses calculated as 100 − banana peel percentage. Differential Evolution was implemented with SciPy using the “best1bin” strategy, a population-size multiplier of 15, a maximum of 1000 generations, a tolerance of 1 × 10^−7^, and final polishing of the best solution. The resulting solutions were summarized using the mean, standard deviation, median, and 2.5th–97.5th percentiles.

Uncertainty associated with the limited number of experimental units was further assessed by reactor-level bootstrap resampling. Five hundred stratified reactor-level bootstrap samples were generated by resampling complete reactor trajectories with replacement within each composition level, preserving the original numbers of reactors per level (two, two, and three, respectively), refitting the model selected pipeline, and repeating the optimization. As a sensitivity analysis, model selection and optimization were also repeated after excluding the temporally truncated 100–0 R3 reactor.

### 2.4. Kinetic Benchmark Modelling

Each reactor trajectory was independently fitted using first-order, modified Gompertz, and modified logistic models. The respective equations were:

(1) $$M\left(t\right)=P\left(1-e^{-kt}\right),$$

(2) $$M\left(t\right)=Pe^{-e^{\left[\frac{R_{m}e}{P}\left(\lambda -t\right)+1\right]}},$$

and

(3) $$M\left(t\right)=\frac{P}{1+e^{\left[\frac{4R_{m}}{P}\left(\lambda -t\right)+2\right]}},$$

where $M\left(t\right)$ is cumulative methane production at time t, P is the estimated asymptotic cumulative methane production, k is the first-order kinetic constant, $R_{m}$ is the maximum methane-production rate, and λ is the lag phase.

Parameters were estimated by bounded nonlinear least squares using the curve_fit function from SciPy. P was constrained between zero and max(3ymax, ymax + 100), k between 0 and 10 h^−1^, *R_m_* between 10^−8^ and max(ymax, 10Rinitial, 1), and Rinitial as max(ymax/(max(tmax, 1)), 0.01) and λ between zero and the maximum observed time of the corresponding reactor. Model performance was described using R^2^, MAE, RMSE, residual sum of squares, and the Akaike information criterion, calculated as AIC = *n* ln(RSS/*n*) + 2*q*, where RSS is the residual sum of squares, *n* is the number of observations and *q* is the number of fitted parameters. The best kinetic model for each reactor was identified by the lowest AIC; in all reactors, this selection agreed with the RMSE ranking. Because the measurements within each trajectory were temporally repeated observations, the kinetic metrics were interpreted as descriptive within-trajectory goodness-of-fit measures and were not directly compared with the LORO-CV performance of selected IA model. All the code can be found in Algorithm S1 of the Supplementary Materials.

## 3. Results and Discussions

### 3.1. Exploratory Predictor Associations

**Figure 1:**
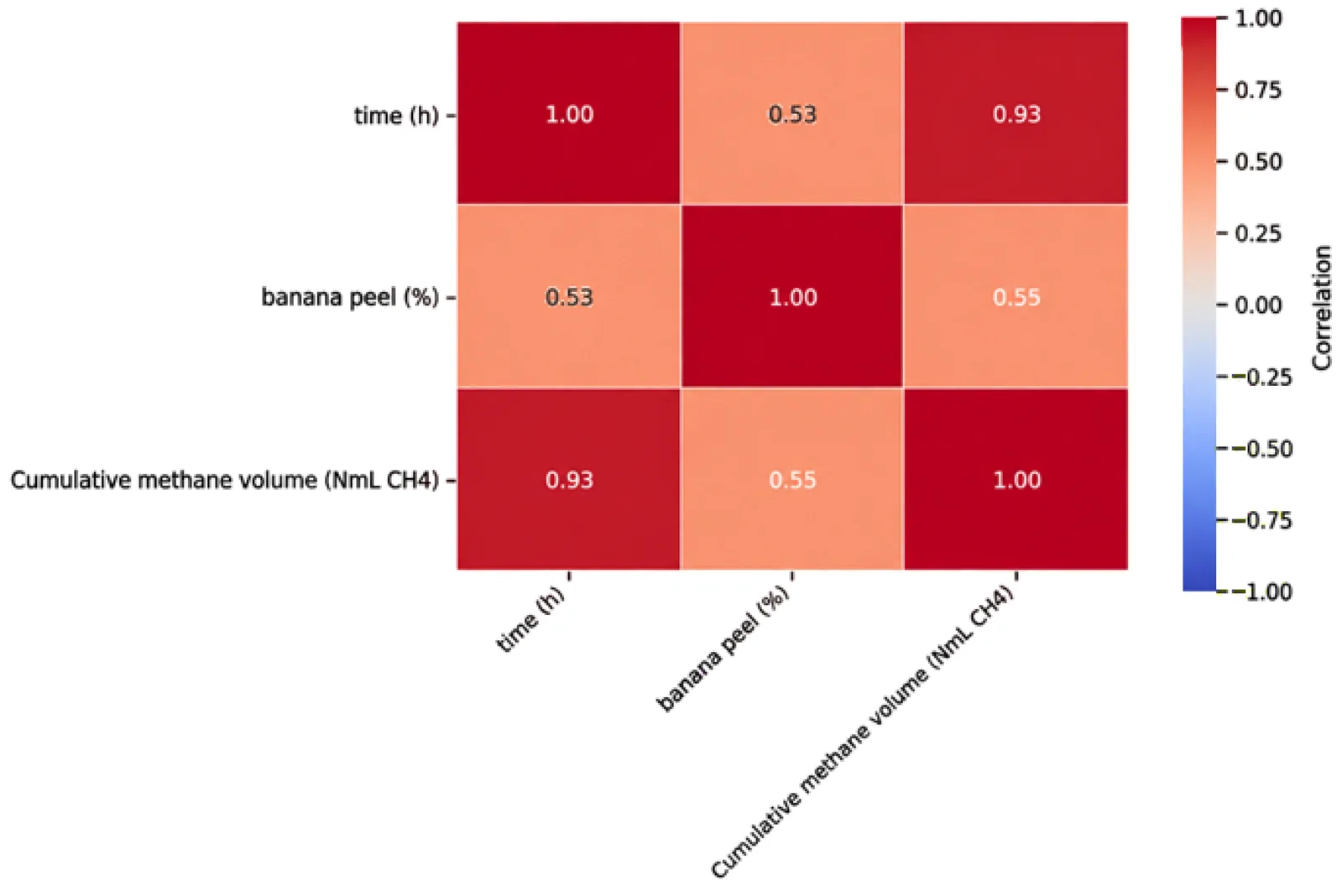
Pearson correlation coefficients among variables.

Digestion time showed the strongest descriptive association with cumulative methane production (Pearson r = 0.934; descriptive F = 33,942.09), followed by banana peel percentage (r = 0.553; descriptive F = 2202.91). Digestion time and banana peel percentage were also positively correlated (Figure 1), reflecting differences in the duration of the experimental trajectories among substrate compositions. The nominal *p*-values of both F statistics were below the numerical reporting precision of the analysis. Nevertheless, because the calculations used repeated observations (Table S1 in Supplementary Materials) within reactors, these statistics were interpreted descriptively and were not used as evidence from independent experimental units.

### 3.2. Reactor-Aware Model Comparison

Under LORO-CV, SVR-RBF obtained the lowest pooled RMSE, therefore, SVR-RBF was selected according to the prespecified primary criterion. Gradient boosting, KNN, random forest, and decision tree produced similar pooled R^2^ values (Table 3).

A factor contributing to the predictive performance is the highly controlled nature of the experimental dataset. Unlike industrial anaerobic digesters, where feedstock variability, environmental disturbances, operational fluctuations, and sensor uncertainty introduce substantial noise, the present experiments were conducted under controlled laboratory conditions using well-defined operating parameters. Consequently, the experimental variability was considerably lower, allowing the ML algorithms to identify relationships among digestion time, substrate composition, and cumulative methane production. Similar observations have been reported in laboratory-scale methane-yield datasets that frequently produce substantially higher prediction accuracies than heterogeneous industrial datasets because the dominant process variables can be measured with greater precision and fewer uncontrolled disturbances [14].

The predictive performance achieved in this study should not be attributed exclusively to the controlled laboratory conditions. Rather, it likely reflects a combination of experimental and modelling factors. The use of standardized batch conditions reduced uncontrolled sources of variability, while the high temporal resolution of methane monitoring provided detailed information on the evolution of cumulative methane production. In addition, digestion time and substrate composition represent process-relevant variables with systematic relationships to methane accumulation, allowing the ML models to capture the nonlinear response patterns present in the experimental data. Previous studies have demonstrated the ability of machine-learning approaches to model the complex nonlinear relationships governing anaerobic digestion and have shown that predictive performance depends on the characteristics of the input data, operating conditions, and model configuration [21].

Methodological aspects should also be considered when comparing performance across studies. Model evaluation was performed using LORO-CV, in which complete reactor trajectories were withheld during validation. Thus, the reported performance represents prediction of unseen reactor trajectories rather than interpolation among timestamped observations from reactors already represented during training. Furthermore, predictor standardization was incorporated within each training fold. Consequently, differences between the performance obtained here and the values reported for more heterogeneous anaerobic digestion datasets likely arise from the combined effects of experimental conditions, dataset characteristics, process variability, and modelling and validation strategies, rather than from laboratory control alone.

Reactor-level paired testing did not establish that SVR-RBF was statistically superior to the alternative models. Although the unadjusted Wilcoxon *p*-values for comparisons with the linear, ridge, and several MLP models were 0.0156, the smallest Holm-adjusted *p*-value was 0.2344 (Table 4). None of the 15 comparisons remained significant at α = 0.05 after multiplicity correction. Thus, SVR-RBF was selected because it minimized the prespecified pooled LORO-CV RMSE, not because statistical superiority over all alternative algorithms was demonstrated.

The group-aware generalization diagnostic for the selected SVR-RBF indicates that the pooled LORO-CV performance does not imply uniform predictive accuracy for every individual reactor. Although SVR-RBF achieved strong pooled LORO-CV performance, its generalization varied among reactors. The diagnostic indicates difficulty reproducing some low-variability or truncated trajectories, whereas the complete banana-peel reactors were predicted more accurately.

### 3.3. Grouped Generalization and SVR Sensitivity

**Figure 2:**
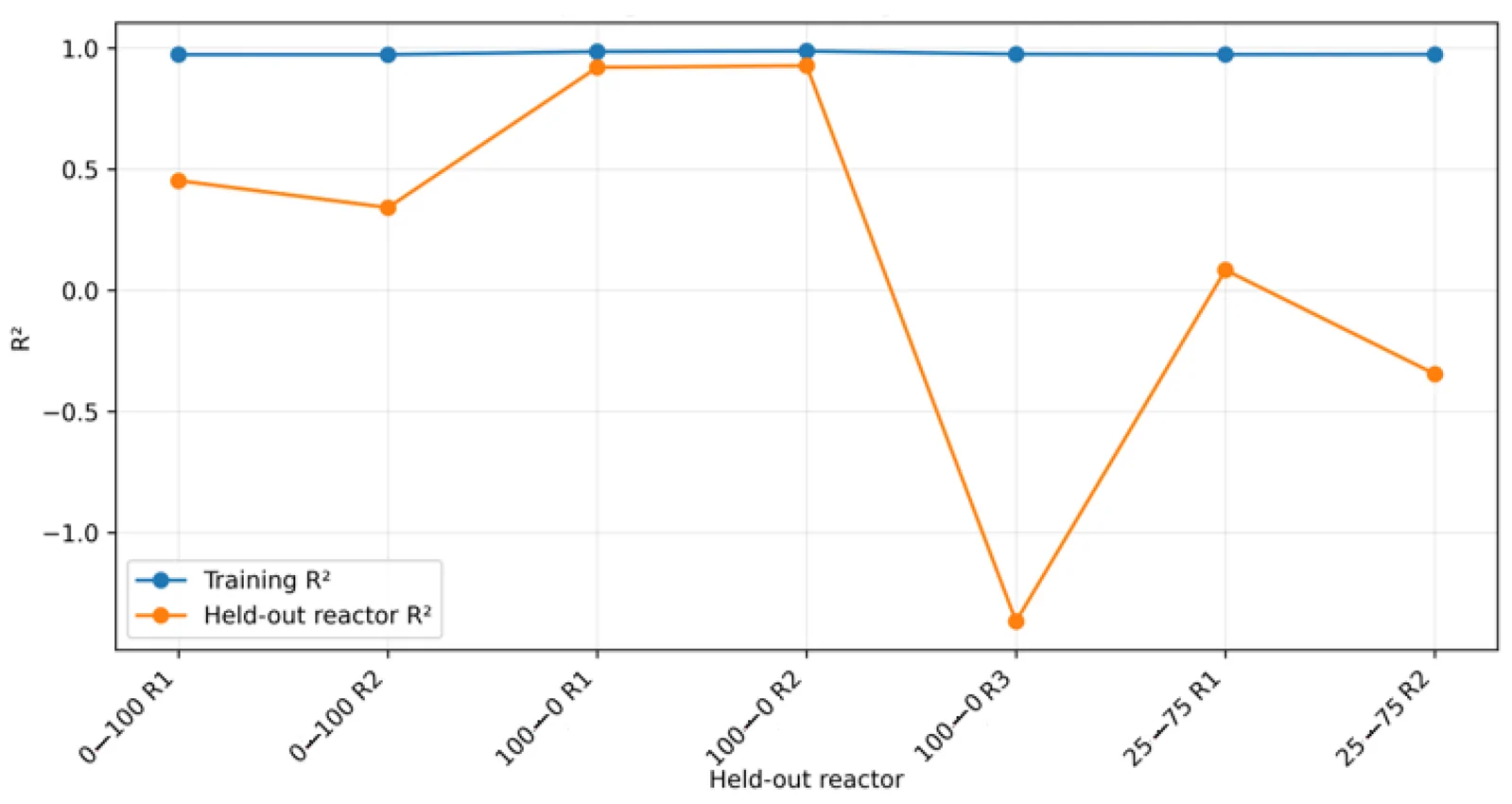
Training and held-out-reactor R^2^ values for SVR-RBF across the seven LORO-CV folds.

Although SVR-RBF achieved a pooled LORO-CV R^2^ of 0.9482, its held-out-reactor R^2^ values varied from −1.366 to 0.928 (Figure 2). These results are not mathematically contradictory because the two calculations evaluate different aspects of predictive performance. The pooled R^2^ was calculated after concatenating predictions from all held-out reactors and therefore reflects the model’s ability to reproduce the overall variation among cumulative methane production trajectories, including the large differences in response magnitude among substrate compositions. In contrast, the reactor-specific R^2^ was calculated using only the variation within each individual trajectory. Consequently, reactors exhibiting an early plateau or a narrow cumulative methane production range had a relatively small total sum of squares, meaning that even modest absolute prediction errors could produce R^2^ values close to zero or below zero. A negative R^2^ indicates that, for that particular reactor, the prediction errors were greater than those obtained by using the reactor mean as a constant prediction; it does not indicate an invalid calculation [22].

The highest held-out R^2^ values were obtained for the two complete banana-peel-only reactors (0.921 and 0.928), whose trajectories covered a broad methane-production range and included the complete sigmoidal development of the process. Conversely, the particularly low value for 100–0 R3 (R^2^ = −1.366) can be attributed partly to its truncated trajectory, which did not cover the extended digestion period or cumulative methane production scale observed in the other banana-peel-only reactors. This interpretation is consistent with the known sensitivity of R^2^ to the variance of the response variable: when the observed range is narrow, relatively small absolute prediction errors can produce low or negative R^2^ values, because the residual sum of squares may exceed the total sum of squares [22].

The lower R^2^ values of the molasses-only and mixed-substrate reactors may likewise reflect their comparatively narrow response ranges, unequal trajectory durations, and differences between replicate trajectories. In addition, methane-production profiles in anaerobic digestion can vary among nominally identical reactors because of differences in substrate degradability, inoculum activity, pH, alkalinity, volatile fatty acids, microbial adaptation, and other biochemical conditions [23,24]. Reviews of machine-learning applications in anaerobic digestion identify limited datasets, heterogeneous operating conditions, and insufficient external validation as important barriers to model generalization [23]. Another study reported higher machine-learning performance during training than during validation with unseen anaerobic-digestion data and attributed part of this difference to feedstock characteristics, temporal variability, and incomplete substrate information [24].

The grouped diagnostic therefore supports the use of LORO-CV rather than invalidating the selected model. Cross-validation strategies that preserve the natural grouping of dependent observations provide a more realistic estimate of performance for unseen experimental units than random partitioning of correlated observations [25]. Accordingly, the pooled LORO-CV metrics were used for comparative model selection, whereas the reactor-specific results were used to delimit model applicability. SVR-RBF can thus be considered a useful surrogate for describing the overall experimental domain and supporting uncertainty-aware optimization, but its predictions should not be interpreted as uniformly accurate for every individual reactor.

A sensitivity analysis for regularization hyperparameter C (1, 10, 50, and 100), kernel coefficient gamma (“scale”, 0.001, 0.01, and 0.1), and epsilon-insensitive loss parameter (0.01, 0.1, and 1.0) under the same LORO-CV scheme was performed. The analysis produced only a marginal improvement when ε was changed from 0.1 to 1.0 while C = 10 and gamma = “scale” were retained. RMSE decreased from 118.087 to 118.046 NmL CH_4_, a difference of approximately 0.035%, while MAE changed from 75.751 to 75.806 NmL CH_4_. This negligible variation indicated that the main conclusion was insensitive to ε within this range. Consequently, the prespecified ε = 0.1 model was retained for the final grouped optimization and bootstrap analysis.

### 3.4. Kinetic Benchmark Analysis

To complement the data-driven modelling framework with physically interpretable kinetic baselines, each cumulative methane trajectory was independently fitted using first-order, modified Gompertz, and modified logistic models, the parameters and the model performances are shown in Table 5.

The kinetic benchmark showed that the temporal cumulative methane production pattern was strongly dependent on substrate composition and could not be adequately represented by a single kinetic formulation across all reactors. First-order kinetics provided the most parsimonious description of the 0/100 molasses-rich trajectories, whereas the modified Gompertz model better captured the rapid sigmoidal behavior observed at 25/75. The modified logistic model was superior for the complete 100/0 banana-peel trajectories, which exhibited a markedly delayed but much larger methane accumulation. Similar composition-dependent differences among first-order, modified Gompertz, logistic, and related kinetic formulations have been reported in batch anaerobic digestion, supporting the use of multiple candidate models rather than assuming that a single kinetic equation is universally appropriate [26].

The modified Gompertz and logistic formulations are particularly useful because their parameters provide interpretable descriptors of batch methane kinetics, including asymptotic methane production, maximum production rate, and lag phase. Recent anaerobic co-digestion studies have similarly used these models to quantify differences in methanogenic response among substrate mixtures and operational conditions [27]. In addition, kinetic modelling of banana-derived residues has shown that Gompertz-type models can closely reproduce cumulative methane or biogas trajectories, confirming their relevance as mechanistic-lean but physically interpretable benchmarks for lignocellulosic agro-industrial substrates [28].

The kinetic models were fitted separately to each observed reactor trajectory and therefore characterize within-reactor degradation behavior, whereas the SVR-RBF was evaluated using leave-one-reactor-out cross-validation and addresses generalization to an unseen reactor trajectory. Consequently, the lower within-curve RMSE values of the kinetic models cannot be interpreted as evidence that they provide better out-of-reactor predictive performance than the SVR. Instead, the kinetic analysis contributes physically interpretable descriptors that are unavailable from the black-box regression model, while the reactor-aware SVR evaluates predictive transfer across experimental trajectories.

The kinetic results also reinforce the need for caution when interpreting the machine-learning optimum. The substantial differences in lag phase, maximum production rate, and asymptotic cumulative methane production among compositions demonstrate that the experimental trajectories represent kinetically distinct regimes. Therefore, the SVR-DE boundary optimum should be interpreted within the experimental domain rather than as evidence of a universal biological optimum.

### 3.5. Process Optimization

Following model validation, the best-performing ML model was integrated with the Differential Evolution algorithm to determine the operating conditions that maximize cumulative methane production within the experimental domain. Across the 70 grouped Differential Evolution runs (see Table S2 in Supplementary Materials), the optimum consistently converged to the upper composition boundary. The median optimal composition was 100% banana peel and 0% sugarcane molasses, with a median digestion time of 310.10 h. The mean optimal time was 316.20 ± 13.54 h, and its 2.5th–97.5th percentile interval was 309.00–348.55 h. Median predicted cumulative methane production was 1433.25 NmL CH_4_, with a corresponding interval of 1325.72–1515.97 NmL CH_4_. Uncertainty in the optimization outcome was evaluated using the seven fold-specific SVR-RBF models, repeated Differential Evolution runs with different random seeds, reactor-level bootstrap resampling, and exclusion of the truncated reactor. Separately, the sensitivity of predictive performance to the main SVR hyperparameters was evaluated under the same LORO-CV procedure (Table 6).

Reactor-level bootstrap resampling (500 iterations) confirmed the stability of the compositional solution, with 99% of bootstrap optima containing ≥99% banana peel. In contrast, considerably wider bootstrap intervals were obtained for optimal digestion time and predicted cumulative methane volume (46.30–366.38 h and 155.68–1531.66 NmL CH_4_, respectively). These results indicate that the direction of the compositional optimum was highly stable, whereas considerably greater uncertainty remained regarding the precise optimal digestion time and magnitude of methane production. Importantly, exclusion of the instrumentally truncated 100–0 R3 trajectory did not alter the optimization conclusion. All sensitivity runs without this reactor again converged to ≥99% banana peel, while the median optimal digestion time (310.20 h) and cumulative methane volume (1431.92 NmL CH_4_) remained consistent with the grouped analysis containing all reactors. This agreement indicates that the boundary solution was not an artifact produced by the incomplete trajectory. The optimization objective maximized cumulative methane volume and did not account for methane-production rate, volumetric productivity, energy consumption, reactor capacity, or economic return. Therefore, the median value of 310.10 h (approximately 12.9 days) represents a model-derived batch endpoint for cumulative methane recovery rather than an operationally or economically optimal digestion time. Although extending digestion may increase cumulative methane recovery, the additional production may not compensate for the associated reduction in reactor throughput and increased capacity and operating requirements.

The convergence of the grouped and bootstrap optimizations toward the banana-peel-rich boundary indicates that the direction of the compositional optimum is robust to reactor resampling, model refitting, and stochastic optimization. However, because the optimum lies at the boundary of the investigated composition domain, it should not be interpreted as evidence that 100% banana peel represents a universal biological optimum. Rather, the results indicate that, within the experimental domain represented by the available reactor trajectories, the SVR-RBF model predicts increasing cumulative methane production toward the banana-peel-rich end of the composition range.

This distinction is particularly relevant when machine-learning models are used for process optimization. Point predictions alone do not characterize the uncertainty associated with model-based decisions, and uncertainty quantification provides additional information regarding the reliability and stability of predicted operating conditions [29,30]. Recent developments in anaerobic-digestion modelling similarly emphasize that model configuration, data preparation, and optimization strategy can materially influence methane-prediction performance and the resulting optimization landscape [31].

Importantly, the reactor-level bootstrap revealed a different degree of uncertainty among the optimized variables. Whereas the composition optimum remained highly stable, with 99% of bootstrap solutions containing ≥99% banana peel, substantially wider intervals were obtained for digestion time and predicted cumulative methane volume. This variability is consistent with the limited number of independent reactor trajectories available for model development and reinforces the need to distinguish between the stability of the location of the compositional boundary optimum and the uncertainty associated with its predicted response magnitude. Consequently, the predicted 100/0 condition should be regarded as a model-supported candidate for subsequent experimental confirmation rather than as a definitively established process optimum.

The boundary solution is biologically plausible in the context of the observed batch trajectories but should not be interpreted as proof of a universally optimal substrate ratio. The methane production potential of banana peel demonstrated in batch digestion studies depends on its non-structural carbohydrate, lignin, hemicellulose, and fibre composition [16,17,28]. Conversely, sugarcane molasses contains readily fermentable carbohydrates that can promote rapid acidogenesis. If acid production exceeds the consumption capacity of syntrophic and methanogenic communities, volatile fatty acids may accumulate and decrease methane formation. Recent solid-state co-digestion experiments with sugarcane molasses reported that increasing molasses proportions caused volatile-fatty-acid accumulation and sharply reduced methane yield, with pure molasses producing the lowest yield among the tested mixtures [18]. This provides a plausible explanation for the comparatively early plateau and low cumulative methane volume of the molasses-rich reactors in the present dataset. However, volatile fatty acids, alkalinity, and temporal pH were not measured in this study; therefore, acidification remains a literature-supported hypothesis rather than an experimentally demonstrated mechanism.

## 4. Conclusions

LORO-CV identified SVR-RBF as the model with the lowest pooled prediction RMSE among the 16 evaluated configurations. Its pooled LORO-CV performance was R^2^ = 0.9482, MAE = 75.75 NmL CH_4_, and RMSE = 118.09 NmL CH_4_. Nevertheless, the reactor-level Wilcoxon tests did not establish statistical superiority after Holm correction, and the grouped diagnostic revealed substantial heterogeneity among held-out reactors. These findings show the importance of separating independent reactors during validation and of reporting reactor-specific performance in addition to pooled metrics. Differential Evolution repeatedly located the maximum predicted cumulative methane production at the experimental boundary of approximately 100% banana peel and 0% molasses. The grouped median optimum was 310.10 h and 1433.25 NmL CH_4_. The composition result remained stable under reactor-level bootstrapping and after exclusion of the truncated reactor, whereas uncertainty in optimal digestion time was considerably greater. Kinetic modelling supported the presence of slower, high-volume methane trajectories in the complete banana-peel-only reactors. The optimized composition is biologically plausible because banana peel is a methanogenic feedstock and high molasses proportions may promote rapid acidogenesis and volatile-fatty-acid accumulation. However, this mechanism was not directly measured. Therefore, the boundary solution is not presented as a universal biological optimum, evidence of co-digestion synergy, or an immediately transferable industrial operating condition. Future experiments should validate compositions near the boundary using additional independent reactors, substrate-normalized and inoculum-blank-corrected methane yields, and measurements of volatile fatty acids, alkalinity, pH, COD, and volatile-solids removal.

## Figures and Tables

**Table 1:** Feedstock’s properties.

Property	Banana Peel	Sugarcane Molasses	Inoculum
COD (g/L)	604.33 ± 6.66	946.67 ± 7.23	247.2 ± 4.58
C/N	20.24 ± 1.01	21.33 ± 2.07	18.63 ± 0.87
Volatile solid (g/L)	633.99 ± 5.64	792.74 ± 0.82	98.22 ± 0.57
TS (g/L)	771.06 ± 0.85	973.26 ± 0.30	125.05 ± 0.50
Carbohydrates (%)	30.07 ± 0.09	32.01 ± 0.09	18.22 ± 0.06
Elemental N (%)	2.04 ± 0.01	1.63 ± 0.16	1.48 ± 0.07
Elemental C (%)	41.16 ± 0.01	34.61 ± 0.08	27.48 ± 0.12
Elemental H (%)	7.12 ± 0.32	6.80 ± 0.47	6.43 ± 0.35
Elemental S (%)	0.41 ± 0.05	1.28 ± 0.37	1.02 ± 0.03

**Table 2:** Experimental composition and inoculum-to-substrate mass ratio of the batch reactors.

Banana Peel/Sugarcane Molasses (%)	Banana Peel (g)	Sugarcane Molasses (g)	Total Substrate (g)	Inoculum (g)
0/100	0.0	50.0	50	100
25/75	12.5	37.5	50	100
100/0	50.0	0.0	50	100

**Table 3:** Model performance based on pooled leave-one-reactor-out predictions.

Model	R^2^_train_	R^2^_LORO_	MAE_LORO_	MSE_LORO_	RMSE_LORO_
SVR-RBF	0.9779	0.9482	75.75	13,944.62	118.09
GPR	0.9884	0.9357	75.36	17,308.18	131.56
GBR	0.9884	0.9332	77.51	17,976.66	134.08
KNN	0.9880	0.9332	77.45	17,978.66	134.08
RF	0.9884	0.9332	77.50	17,983.44	134.10
DT	0.9879	0.9325	79.16	18,176.00	134.82
MLP_6	0.9834	0.9280	84.34	19,372.66	139.19
MLP_7	0.9836	0.9276	85.37	19,502.36	139.65
MLP_8	0.9861	0.9229	85.84	20,765.35	144.10
MLP_1	0.9261	0.8869	125.77	30,455.76	174.52
MLP_3	0.9360	0.8744	131.42	33,823.31	183.91
MLP_2	0.9353	0.8733	128.23	34,112.64	184.70
MLP_4	0.9421	0.8724	124.84	34,358.79	185.36
MLP_5	0.9383	0.8646	118.98	36,443.46	190.90
Linear	0.8757	0.8449	168.86	41,744.91	204.32
Ridge	0.8757	0.8449	168.86	41,745.33	204.32

**Table 4:** Reactor-level Wilcoxon signed-rank comparisons between SVR-RBF and the alternative models, with Holm-adjusted *p*-values.

Compared Model	*p* Value	*p* Value Holm
Linear	0.0156	0.2344
Ridge	0.0156	0.2344
MLP_1	0.0156	0.2344
MLP_2	0.0156	0.2344
MLP_3	0.0156	0.2344
MLP_4	0.0156	0.2344
MLP_5	0.0469	0.4219
RF	0.2188	1
GBR	0.2188	1
KNN	0.2188	1
GPR	0.2969	1
DT	0.8125	1
MLP_6	0.8125	1
MLP_7	0.8125	1
MLP_8	0.9375	1

**Table 5:** Best-fitting kinetic model and estimated parameters for each experimental reactor.

Reactor	Best Kinetic Model	P (NmL CH_4_)	k (h^−1^)	$R_{m}$ (NmL CH_4_ h^−1^)	λ	R^2^	RMSE
0–100 R1	First-order	131.90	0.163	-	-	0.819	9.06
0–100 R2	First-order	127.96	0.163	-	-	0.830	8.20
25–75 R1	Modified Gompertz	139.27	-	52.99	1.24	0.958	3.81
25–75 R2	Modified Gompertz	127.09	-	55.01	1.35	0.968	3.60
100–0 R1	Modified Logistic	1512.86	-	13.70	135.32	0.983	79.08
100–0 R2	Modified Logistic	1318.82	-	8.89	98.91	0.976	79.21
100–0 R3 *	Modified Logistic	124.90	-	55.13	2.04	0.998	2.15

* 100/0 R3 was instrumentally truncated and its kinetic parameters are reported descriptively but were not interpreted as representative of a complete methane-production trajectory.

**Table 6:** Uncertainty and stability of the predicted optimum.

Analysis	Optimal Banana Peel (%)	Optimal Digestion Time (h)	Predicted Cumulative Methane Volume (NmL CH_4_)
Grouped LORO-DE, median	100.00	310.10	1433.25
Empirical 2.5–97.5% interval	≈100.00	309.00–348.55	1325.72–1515.97
Reactor-level bootstrap, median	100.00	324.76	1435.63
Bootstrap 2.5–97.5% interval	99.49–100.00	46.30–366.38	155.68–1531.66

## Data Availability

The original contributions presented in this study are included in the Supplementary Material. Further inquiries can be directed to the corresponding author.

## Supplementary Materials

The following supporting information can be downloaded at: https://www.mdpi.com/article/10.3390/foods15173161/s1, Table S1: Experimental data; Table S2: Experimental results; Algorithm S1: Modelling and optimization code.

## Abbreviations

The following abbreviations are used in this manuscript:

AD	Anaerobic digestion
ML	Machine learning
COD	Chemical oxygen demand
LR	Linear Regression
SVR-RBF	Support Vector Regression with a Radial Basis Function
RF	Random Forest
DT	Decision Tree
GBR	Gradient Boosting Regression
KNN	K-Nearest Neighbors Regression
GPR	Gaussian Process Regression
MLP	Multilayer Perceptron
R^2^	Coefficient of determination
MAE	Mean absolute error
MSE	mean squared error
RMSE	Root mean squared error
AIC	Akaike Information Criterion
LORO-CV	Leave-one-reactor-out cross-validation
